# Effects of High Glucose on Vascular Endothelial Growth Factor Synthesis and Secretion in Aortic Vascular Smooth Muscle Cells from Obese and Lean Zucker Rats

**DOI:** 10.3390/ijms13089478

**Published:** 2012-07-26

**Authors:** Gabriella Doronzo, Michela Viretto, Isabella Russo, Luigi Mattiello, Giovanni Anfossi, Mariella Trovati

**Affiliations:** Internal Medicine and Metabolic Disease Unit, Department of Clinical and Biological Sciences, San Luigi Gonzaga Faculty of Medicine of the Turin University, San Luigi Gonzaga Hospital, Orbassano (Turin), 10043, Italy; E-Mails: michela.viretto@unito.it (M.V.); isabella.russo@unito.it (I.R.); luigi.mattiello@unito.it (L.M.); mariella.trovati@unito.it (M.T.)

**Keywords:** vascular endothelial growth factor, vascular smooth muscle cells, glucose, osmotic stress, insulin resistance, diabetes, lean zucker rats, obese zucker rats

## Abstract

Type 1 diabetes is characterized by insulin deficiency, type 2 by both insulin deficiency and insulin resistance: in both conditions, hyperglycaemia is accompanied by an increased cardiovascular risk, due to increased atherosclerotic plaque formation/instabilization and impaired collateral vessel formation. An important factor in these phenomena is the Vascular Endothelial Growth Factor (VEGF), a molecule produced also by Vascular Smooth Muscle Cells (VSMC). We aimed at evaluating the role of high glucose on VEGF-A_164_ synthesis and secretion in VSMC from lean insulin-sensitive and obese insulin-resistant Zucker rats (LZR and OZR). In cultured aortic VSMC from LZR and OZR incubated for 24 h with d-glucose (5.5, 15 and 25 mM) or with the osmotic controls l-glucose and mannitol, we measured VEGF-A_164_ synthesis (western, blotting) and secretion (western blotting and ELISA). We observed that: (i) d-glucose dose-dependently increases VEGF-A_164_ synthesis and secretion in VSMC from LZR and OZR (*n* = 6, ANOVA *p* = 0.002–0.0001); (ii) all the effects of 15 and 25 mM d-glucose are attenuated in VSMC from OZR *vs.* LZR (*p* = 0.0001); (iii) l-glucose and mannitol reproduce the VEGF-A_164_ modulation induced by d-glucose in VSMC from both LZR and OZR. Thus, glucose increases via an osmotic mechanism VEGF synthesis and secretion in VSMC, an effect attenuated in the presence of insulin resistance.

## 1. Introduction

Diabetes Mellitus is a relevant cause of cardiovascular morbility and mortality: both type 1 diabetes, resulting from insulin deficiency, and type 2 diabetes, characterized by the coexistence of insulin resistance and inadequate insulin secretion, present both microvascular (*i.e.*, retinopathy and nephropathy) and macrovascular (*i.e*., atherosclerotic coronary, cerebral and peripheral vascular disease) complications [1]. Among the mechanisms involved in diabetic micro-angiopathy and macro-angiopathy, a peculiar role is played by the altered synthesis and secretion of proteins of the Vascular Endothelial Growth Factor (VEGF) family [1].

VEGF family includes different molecules, and in particular VEGF-A, a glycoprotein with five distinct isoforms, resulting from alternative exon splicing of a single gene [2]. VEGF-A shows mitogenic and survival effects on vascular endothelial cells and it is involved in different vascular processes. In particular, VEGF-A plays a role in: (i) physiological and pathological angiogenesis, *i.e.*, sprouting of new vessels from the existing-ones, a process involved in embryonic development, reproductive functions, wound and fracture healing, post-ischaemic cardiovascular and cerebrovascular revascularization, proliferative retinopathies, and in age-associated and tumor-associated neovascularization; (ii) arteriogenesis, *i.e.*, maturation or de novo growth of collateral vessels, mainly occurring as a compensatory mechanism after vessel occlusion [3–5].

Since VEGF-A is not only a survival factor for endothelial cells, but also a regulator of microvascular permeability and a potent vasodilator, it is considered a key molecule in renal and retinal capillary function [2]. It also promotes the formation of post-ischemic collateral vessels and plays a role in wound healing [2]. For all these reasons, changes in synthesis and secretion of VEGF-A are strongly correlated with diabetic vascular complications.

Ischemia is not the only stimulus for VEGF synthesis: actually, many molecules such as cytokines and growth factors are involved in the regulation of VEGF-A synthesis and secretion in different cell types. In particular Vascular Smooth Muscle Cells (VSMC), which play a crucial role in angiogenesis and arteriogenesis, synthesize and secrete VEGF-A [3–5]. In our laboratory, we previously demonstrated that in VSMC obtained as primary culture from the aorta of lean, insulin sensitive Zucker fa/+ rats, insulin stimulates VEGF-A_164_ protein synthesis and secretion [6,7], and that this insulin action is blunted in VSMC from the aorta of obese, insulin-resistant Zucker fa/fa rats, a classical animal model of insulin resistance [6,7]. These results explain, at least in part, why insulin resistant states are characterized by a poor collateral vessel formation, also independently of diabetes mellitus [8]. However, diabetes too is characterized by an impaired formation of collateral vessels, playing a role in the pathogenesis of cardiovascular events [9–11].

As it is well known, the main feature characterizing diabetes mellitus is hyperglycemia, which plays a pivotal role in the pathogenesis of diabetes vascular complications [12]: hyperglycemia, in particular, modifies VEGF synthesis and secretion in different cell types, such as glomerular podocytes [13], mesangial cells [14,15] and the retinal endothelial cells [16,17]. Only two studies addressed so far the high glucose modulation of VEGF in VSMC: both of them investigated the influence of incubation with high glucose (25 mM) for several VSMC passages in culture [18,19]. Natarajan *et al.* showed that high glucose increases mRNA and protein expression of VEGF and VEGF secretion in human and porcine aortic VSMC with a mechanism independent by the osmotic stress since it is not reproduced by mannitol [18], whereas Dulak *et al.* did not observe an influence of high glucose on VEGF secretion in VSMC from rat thoracic aorta [19]. So far, no study has evaluated the influence of a relatively short-time incubation with high glucose in VSMC at the same culture passage. Furthermore, it is not known whether insulin resistance affects the responses of VSMC to high glucose.

The aim of the present study is to evaluate whether a 24 h incubation with high glucose influences synthesis and secretion of VEGF-A_164_ in cultured aortic VSMC from insulin sensitive lean Zucker rats and insulin resistant obese Zucker rats and whether osmotic stress is involved in this putative phenomenon.

## 2. Results and Discussion

### 2.1. Effects of High Glucose on VEGF-A_164_ Synthesis in Aortic VSMC from Insulin Sensitive LZR and Insulin Resistant OZR

**Figure 1:**
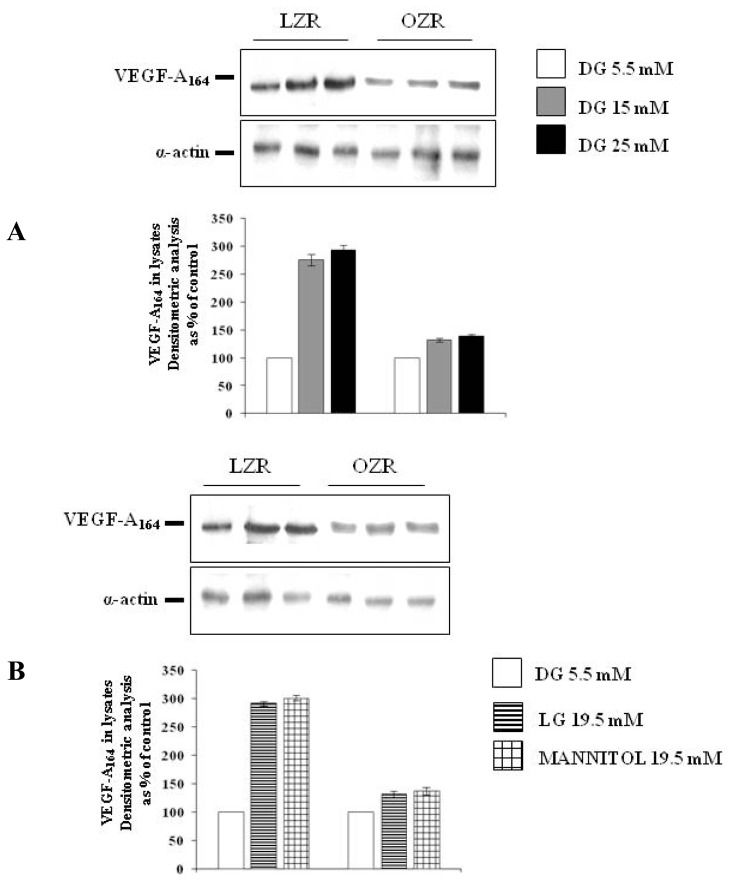
(**A**) Dose-dependent effects on Vascular Endothelial Growth Factor (VEGF)-A_164_ synthesis (Western immunoblotting and its densitometric analysis) elicited by a 24 h of incubation with d-glucose (5.5, 15 and 25 mM) in Vascular Smooth Muscle Cells (VSMC) from lean insulin-sensitive Zucker fa/+ rats (LZR) and obese insulin-resistant Zucker fa/fa rats (OZR); (**B**) Role on VEGF-A_164_ synthesis (Western immunoblotting and its densitometric analysis) elicited in VSMC from LZR and OZR by a 24 h of incubation with 19.5 mM l-glucose or mannitol, *i.e.*, with a concentration iso-osmolar to 25 mM d-glucose, taking into account the presence of d-glucose 5.5 mM in the culture medium. Statistical analysis is described in the text.

As shown in Figure 1 panel A, in aortic VSMC from LZR a 24 h incubation with d-glucose increased VEGF-A_164_ synthesis (*n* = 6, ANOVA *p* = 0.0001), all the concentrations tested exerting a significant effect (*p* < 0.05 with Bonferroni’s analysis). In aortic VSMC from OZR the effects of d-glucose were already present (ANOVA, *p* = 0.0001), but attenuated in comparison with those observed in VSMC from LZR (*n* = 6, *p* = 0.0001 both at 15 and at 25 mM d-glucose).

### 2.2. Effects of High Glucose on VEGF-A_164_ Secretion in Aortic VSMC from Insulin Sensitive LZR and Insulin Resistant OZR

**Figure 2:**
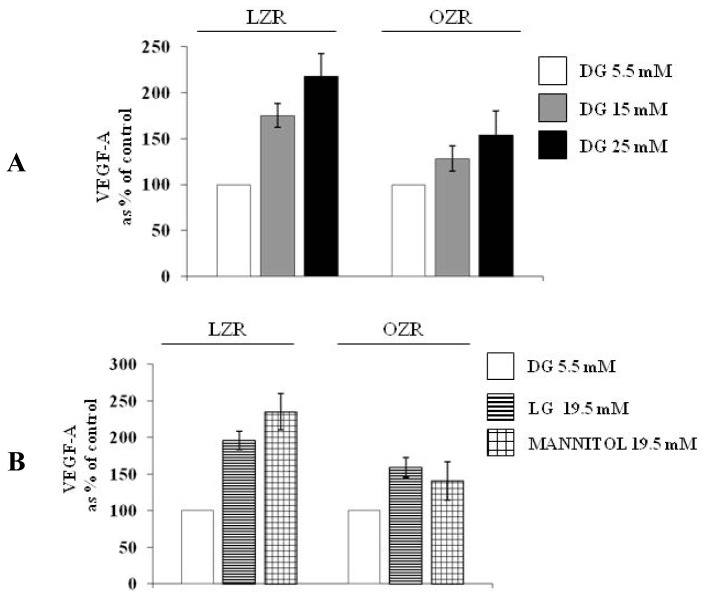
(**A**) Dose-dependent effects on VEGF-A (ELISA) elicited by a 24 h incubation with d-glucose (5.5, 15 and 25 mM) in VSMC from LZR and OZR; (**B**) Role on VEGF-A secretion (ELISA) elicited in VSMC from LZR and OZR by a 24 h incubation with 19.5 mM l-glucose or mannitol, *i.e.*, with a concentration iso-osmolar to 25 mM d-glucose, taking into account the presence of d-glucose 5.5 mM in the culture medium. Statistical analysis is described in the text.

**Figure 3:**
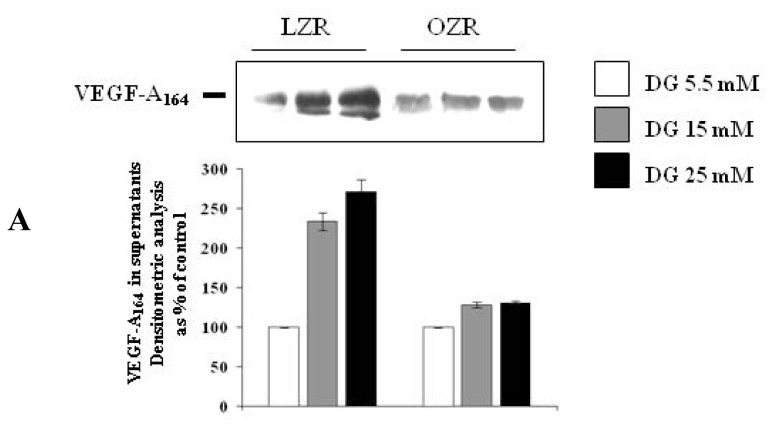

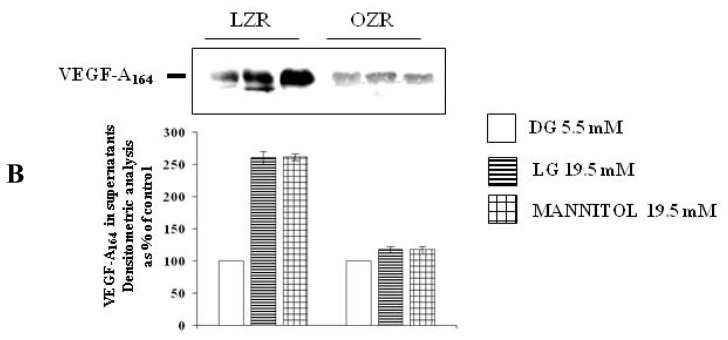
(**A**) Dose-dependent effects on VEGF-A_164_ secretion (Western immune blotting and its densitometric analysis) elicited by a 24 h of incubation with d-glucose (5.5, 15 and 25 mM) in VSMC from LZR and OZR.; (**B**) Effects on VEGF-A_164_ secretion (Western immunoblotting and its densitometric analysis) elicited by a 24 h incubation with 19.5 mM l-glucose or mannitol in VSMC from LZR and OZR, *i.e.*, with a concentration iso-osmolar to 25 mM d-glucose, taking into account the presence of d-glucose 5.5 mM in the culture medium. Statistical analysis is described in the text.

VEGF-A_164_ secretion has been evaluated via both ELISA and western blotting (Figures 2 and 3, Panel A). We observed that: (i) in aortic VSMC from LZR, a 24 h incubation with d-glucose increased in a concentration dependent manner VEGF-A_164_ secretion (*n* = 6, ANOVA *p* = 0.003 for ELISA and *p* = 0.0001 for western blotting, *p* < 0.05 with Bonferroni’s analysis); (ii) in aortic VSMC from OZR, the actions of d-glucose on VSMC secretion were already present (*n* = 6, ANOVA *p* = 0.0001 for ELISA and *p* = 0.002 for western blotting), but were attenuated in comparison with those observed in VSMC from LZR (*n* = 6, *p* = 0.0001 at 15 mM and 25 mM of d-glucose both for ELISA and for western blotting).

### 2.3. Role of Osmotic Stress

As shown in Figures 1–3 panels B, the effects of d-glucose on VEGF-A_164_ synthesis and secretion were reproduced by both l-glucose and mannitol in VSMC from LZR (*n* = 6, *p* = 0.0001 for both, ns *vs.* 25 mM d-glucose).

Also in VSMC from OZR the effects of d-glucose were reproduced by both l-glucose and mannitol (*n* = 6, ns *vs.* d-glucose 25 mM): as described for d-glucose (Figures 1–3, panels A), the effects of both l-glucose and mannitol were lower than those observed in VSMC from LZR (*n* = 6, *p* = 0.0001 for both).

## 3. Experimental Section

### 3.1. Study Design

To evaluate the effects of high glucose on VEGF-A_164_ protein synthesis and secretion in conditions of insulin sensitivity and of insulin resistance, aortic VSMC from lean insulin-sensitive Zucker fa/+ rats (LZR) and from obese insulin-resistant Zucker fa/fa rats (OZR) were incubated for 24 h: (i) in normal glucose, *i.e.*, with the d-glucose concentration of 5.5 mM present in the Minimal Essential Medium (MEM) (*n* = 6); (ii) in high glucose, *i.e.*, with d-glucose at final concentrations of 15 and 25 mM (*n* = 6).

To evaluate whether the effects of high glucose are attributable to the osmotic stress, VSMC from aortas of LZR and OZR were incubated for 24 h with 19.5 mM of the two osmotic controls l-glucose (*n* = 6) and mannitol (*n* = 6), a concentrations chosen to reproduce the osmolarity characterizing the experiments with 25 mmol/L d-glucose, obtained by adding 19.5 mM d-glucose to the 5.5 mM d-glucose already existing in the medium.

### 3.2. Chemicals

d-glucose, l-glucose and mannitol were purchased from Sigma-Aldrich (St. Louis, MO, USA). Compounds used for cell culture, western blotting and ELISA are detailed in the specific paragraphs.

### 3.3. Cell Culture and Characterization

All the experiments were carried out with VSMC obtained in primary culture in our laboratory from aortas of LRZ and OZR. Rats, purchased from Charles River Laboratories Italy (Calco, Italy), were fed with standard rodent chow and water ad libitum until 14 weeks old and then sacrificed with CO_2_ after a 12-h fast. Aortas were removed immediately after sacrifice and VSMC isolation and characterization were made according to classical procedures, as previously reported [6]. Briefly, VSMC were cultured in MEM supplemented with 10% fetal calf serum (FCS), 100 U/mL of penicillin, 100 μg/mL of streptomycin, 10 mM of glutamine and vitamins, and buffered with 10 mM of *N*-tris (hydroxymethyl) methyl-2 aminoethane-sulphonic acid (TES) and 10 mM of *N*-(2-hydroxyethyl) piperazine-N1-2-ethanesulphonic acid (HEPES). Incubation was carried out at 37 °C in a humidified incubator with an atmosphere of 95% O_2_:5% CO_2_, and medium was replaced with fresh medium every 4 days. Purity of cultures was evaluated by immunofluorescence staining and by Fluorescence Activated Cell Sorting (FACS) separation in VSMC incubated with: (i) a monoclonal antibody anti α-smooth muscle actin (Sigma-Aldrich), the specific marker of smooth muscle cells; (ii) monoclonal antibodies anti specific markers for endothelial cells, *i.e.*, von Willebrand Factor (Dako, Carpinteria, CA, USA) and CD31 (Serotec, Oxford, UK), to exclude the presence of endothelial cells in the cultures; (iii) a fluorescein isothiocyanate (FITC)-conjugated secondary antibody goat antimouse immunoglobulins (Dako, Carpinteria, CA, USA). Fixed cells were analyzed with fluorescence a LSM Zeiss confocal microscope (Carl Zeiss, Jena GmbH, Jena, Germany). Antigen expression was also analyzed with a FACS scan flow cytometer (Becton Dickinson, Milan, Italy). With FACS separation, 87%–99% of the cells were positive for α-actin and less than 1% were positive for CD31 and von Willebrand factor.

For the experiments, VSMC were used at the 6–7th passage and cultured in MEM with 10% FCS until 80% confluence was achieved. Then, MEM with 10% FCS was removed and cells were cultured overnight in medium with 1% FCS, that was changed before the experiments.

### 3.4. VEGF Synthesis and Secretion

To evaluate VEGF synthesis, at the end of the incubation times, VEGF protein expression was measured by western blots. Briefly, VSMC were washed two times with phosphate buffered saline (PBS) and then solubilized with boiling Laemmli buffer addictioned with a proteinase inhibitor cocktail (Sigma-Aldrich). Then, lysates were centrifuged at 13,000 rpm for 10 min and the protein concentration was determined by the Bradford Reagent method (Sigma-Aldrich). Samples of cell lysates and supernatants were separated by 10% sodium-dodecylsulphate-polyacrylamide gel electrophoresis (SDS-PAGE) and transferred to Immobilon-P Transfer Membrane (Millipore Corporation, Bedford, MA, USA). Membranes have been incubated for 60 min with a monoclonal antibody (antibody dilution 1:1000) against VEGF-A (Santa Cruz Biotechnology Inc, CA, USA) in PBS containing 0.1% Tween-20 (Sigma-Aldrich). Then, blots have been incubated with a peroxidase-conjugate affine pure goat anti mouse IgG antibody (antibodies dilutions was 1:10,000) (Jackson Immunoresearch Laboratories, West Grove, PA, USA) for 45 min in PBS containing 0.1% Tween-20 (Sigma-Aldrich). As a loading control, we used a monoclonal antibody anti-α-smooth muscle actin (Santa Cruz Biotechnology Inc, CA, USA). After washing, proteins have been detected with ECL-plus kit (Amersham Pharmacia Biotech, Sunnyvale, CA, USA). Blots have been analysed densitometrically by using the image analyzer Kodak 1D Image Analysis Software. Density of bands has been quantified as arbitrary units and changes in protein synthesis have been expressed as percent of the density measured at d-glucose 5.5 mM.

To evaluate VEGF-A secretion, at the end of the incubation times the VSMC supernatant-conditioned medium was stored at −20°C. Then, VEGF-A secretion was measured both with protein expression using western blotting technique as detailed above in the section concerning VEGF-A synthesis and by a specific enzyme-linked immunosorbent assay (ELISA) kit from R & D System (Minneapolis, MN, USA). VEGF-A protein concentrations in the supernatants were normalized to the total amount of cell proteins contained in each dish and expressed as concentrations as percent of the concentration measured at d-glucose 5.5 mM. Protein concentrations were determined by Bradford Reagent method (Sigma-Aldrich).

### 3.5. Statistical Analysis

All the experiments were carried in sextuplicate. Data are expressed as mean ± SEM. Significance was evaluated, when appropriate, by unpaired Student’s *t*-test and by one-way ANOVA followed by the Bonferroni’s analysis. A *p* < 0.05 was considered significant.

## 4. Conclusions

This study shows that a 24 h incubation with high glucose increases via osmotic stress VEGF-A_164_ synthesis and secretion in cultured rat aortic VSMC, and that this effect is deeply attenuated in the presence of insulin resistance. As far as we know, this is the first demonstration of a glucose regulation of VEGF-A_164_ in VSMC obtained by a relatively short cell incubation, since the only previous study demonstrating a stimulating role of high glucose on VEGF synthesis and secretion was carried out by incubating VSMC with glucose from at least two passages, and therefore for many days [18]. The relatively rapid effects of high glucose on VEGF synthesis and secretion in VSMC support the hypothesis that *in vivo* glucose fluctuations, and not only chronic hyperglycaemia, can influence this angiogenetic molecule. Owing to the different experimental conditions, it is not surprising that the mechanisms involved are different, the osmotic stress playing a role only when incubation with glucose is relatively short as in our study.

Furthermore, our study originally shows that high glucose modulation of VEGF-A_164_ is deeply impaired in VSMC from insulin-resistant rats.

Literature shows that short-time incubations with high glucose increase VEGF synthesis and secretion in glomerular podocytes [13], in mesangial cells [14,15] and in the retinal endothelial cells [16,17] thus, results we obtained in VSMC are in keeping with those obtained in other cell types.

The role of osmotic stress as a mediator of high glucose actions has not been observed before either in VSMC or in other cell types [13–18] in our study, it is confirmed by two different molecules employed as osmotic controls, *i.e.*, l-glucose and mannitol, both for VEGF synthesis and for VEGF secretion.

As far as we know, we are providing here the first evidence that both high glucose and iso-osmotic compounds modulation of VEGF-A_164_ is attenuated in the presence of insulin resistance: actually, the effects of d-glucose, mannitol and l-glucose are lower in VSMC from OZR *vs.* LZR. We previously observed that insulin and nitric oxide induction of VEGF-A_164_ synthesis and secretion are reduced in VSMC from OZR [6,7].

The biological roles of VEGF differ in different cell types [1]. For instance, in the eye VEGF promotes both retinal and choroidal neovascularization, which characterizes the more severe form of diabetic retinopathy, responsible for visual loss in this case, the stimulating effects of high glucose on VEGF are deeply involved in the progression of diabetic retinopathy until the last stages [20]. In kidney, VEGF plays a critical role in maintaining normal glomerular podocyte function and is a key regulator of permeability: in the presence of diabetes mellitus, and therefore in a condition of chronic hyperglycaemia, elevated VEGF concentrations increase glomerular permeability and contribute to the development of diabetic nephropathy [21].

But what is the biological meaning of VEGF synthesis in VSMC and of its stimulation by high glucose? VEGF produced by VSMC can play a role both in post-ischemic collateral vessel formation and in the neovascularization of the atherosclerotic plaque. At the plaque level, in particular, neovascularization exerts both beneficial actions by prevention of cell death due to the increased supply of oxygen and nutrients leading to plaque growth and stabilization and deleterious ones, by causing intraplaque hemorrhage and plaque rupture, the precipitating factor of acute vascular events [22]. As previously reviewed VEGF influences the growth and fate of the atherosclerotic plaques [23]. Thus induced by high glucose, VEGF released from VSMC is both protective and dangerous and its final effect can change in different clinical settings. For instance, the data of the present study support the hypothesis that the effects of high glucose on VEGF in VSMC could be different in type 1 diabetes, which is mainly insulin deficient, and in type 2 diabetes, where a relative insulin deficiency is commonly accompanied by a profound insulin resistance, since the latter seems to attenuate the glucose effects on VEGF.
